# Artificial Intelligence Applications to Public Health Nutrition

**DOI:** 10.3390/nu15194285

**Published:** 2023-10-08

**Authors:** Ruopeng An, Xiaoxin Wang

**Affiliations:** 1 Brown School, Washington University, St. Louis, MO 63130, USA; 2 Department of Physical Education, Tsinghua University, Beijing 100190, China; wang.xiaoxin@wustl.edu

Public health nutrition occupies a paramount position in the overarching domains of health promotion and disease prevention, setting itself apart from nutritional investigations concentrated at the individual level. While the latter delves deep into the nuances of individual dietary needs, metabolic responses, and genetic predispositions, public health nutrition aims to understand and influence the dietary behaviors of entire populations. Such research spans a gamut of areas: from examining the intricate interplay of food supply and the broader food environment [1,2] and analyzing the implications of government interventions such as soda taxes and healthy food subsidies [3,4], to evaluating how economic conditions and climate change impact food access and choices [5,6]. In an increasingly globalized world, the challenges of accurately measuring dietary intakes at a large scale are also being addressed with innovative methodologies and tools [7,8]. The collective aim of these endeavors is not merely to ensure that individuals eat healthily but to create an environment where the healthier choice becomes easier for everyone.

Artificial intelligence (AI) has witnessed an unprecedented acceleration in capabilities and adoption in recent years. This rapid advancement, driven by innovations in machine learning algorithms and the exponential growth in computational power, has permeated and reshaped a myriad of human activities and social sectors [9]. In the domain of public health and nutrition, AI’s potential is being realized in various innovative ways. AI algorithms have been instrumental in mapping and analyzing food environments, identifying areas with limited access to nutritious foods, often termed “food deserts” [10]. Machine learning models have also been utilized to predict the effects of potential policy interventions, such as how specific taxation or subsidies might impact population dietary habits [11]. At a larger scale, AI has assisted in monitoring global food supply chains, ensuring food safety, and predicting potential disruptions due to climate change [12,13]. With the vast amount of data now available, ranging from satellite imagery of agricultural regions to social media discussions on dietary habits, AI provides tools to synthesize and interpret these data in ways that can inform public health nutrition strategies and interventions [14,15,16].

The potential applications of AI in public health nutrition are vast, and the current body of research may only scratch the surface of what is achievable. The richness of data and the evolving capabilities of AI offer a myriad of possibilities that are waiting to be explored. We encourage our fellow researchers to think beyond the traditional boundaries and explore innovative ways to harness the power of AI in promoting healthier diets and improving nutritional health at the population level. Imagine a world where AI-driven models predict the nutritional needs of entire regions based on climate, soil quality, and socio-economic factors, helping policymakers prioritize food production and distribution. Consider the potential of AI tools that can monitor global food trends, identify emerging patterns, and give public health officials a head start in formulating interventions, or even the possibility of AI systems collaborating across countries to harmonize nutritional guidelines, ensuring that a consistent message reaches all populations.

However, with great power comes great responsibility. There are significant concerns with AI technology that cannot be overlooked. Data privacy is paramount, and we must ensure that any AI-driven initiative in public health respects individual and community rights. Bias is another major concern; algorithms are only as good as the data on which they are trained, and any bias in that data can lead to biased outcomes that could exacerbate existing health disparities. We encourage the community not only to leverage AI’s strengths but also to develop innovative ways to mitigate its potentially harmful effects. This is an era of collaboration between technology and public health, and together, we can ensure that AI serves as a force for good in promoting nutritional health worldwide.
